# Heterogeneous Wettability Alters Methane Migration and Leakage in Shallow Aquifers

**DOI:** 10.1021/acs.est.5c15451

**Published:** 2026-03-27

**Authors:** Sabber Khandoozi, Siddharth Gautam, Craig Dietsch, Muhammad Sahimi, David Cole, Reza Soltanian

**Affiliations:** † Department of Geosciences, 2514 University of Cincinnati, Cincinnati, Ohio 45221, United States; ‡ School of Earth Sciences, 2647 The Ohio State University, Columbus, Ohio 43210, United States; § Mork Family Department of Chemical Engineering and Materials Science, 5116 University of Southern California, California 90089-1211, United States; ∥ Department of Environmental Engineering, 2514 University of Cincinnati, Cincinnati, Ohio 45221, United States

**Keywords:** fugitive methane leakage, capillary heterogeneity, heterogeneous wettability, molecular dynamics, atmospheric methane emissions

## Abstract

Capillary heterogeneity is increasingly recognized as a first-order control of gas plume migration and trapping in aquifers and storage formations. We show that spatial variability in the water–methane contact angle, set by mineralogy and salinity, reshapes capillary entry pressures and, in turn, migration pathways. Using molecular dynamics simulation, we estimate contact angles on quartz and kaolinite under fresh and saline conditions and embed these results in continuum-scale multiphase flow simulations via a contact-angle-informed Leverett *J*-function, mapping wettability directly onto the flow properties at continuum scale. Accounting for contact-angle heterogeneity changes the methane behavior: mobile and residually trapped methane in aquifers decrease by up to 10%, while leakage to the atmosphere increases by as much as 20%. The magnitude of this effect is scaled with the permeability contrast, leakage rate, salinity, and facies proportions. By coupling molecular-scale wettability to continuum-scale flow and transport, the cross-scale framework provides a more physically grounded basis for groundwater protection and risk assessments and yields more reliable emissions estimates. The approach can be generalized to other subsurface gas transport problems including hydrogen and carbon dioxide storage as well as natural releases such as methane from permafrost thaw.

## Graphical abstract

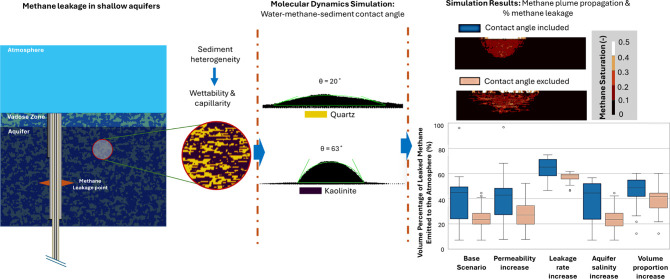

## 1 Introduction

Methane (CH_4_) leakage from subsurface sources into shallow aquifers and the atmosphere is a significant environmental concern.^1–4^ Fugitive CH_4_ may originate from compromised wells, natural faults, fractures, or surface activities and is further complicated by naturally occurring biogenic CH_4_.^5–11^ As a potent greenhouse gas, CH_4_ poses risks to groundwater quality, climate, and public safety, making it essential to predict its migration pathways.^12–15^ Recent work highlights the strong influence of small-scale geologic features, with capillary heterogeneity identified as a primary controlling factor for plume migration and trapping.^16,17^ Neglecting capillary pressure variability biases predictions, often overstating atmospheric emissions and understating aquifer retention.^16,18^ Building on this foundation, we investigate how mineral- and salinity-dependent wettability, expressed as water–CH_4_ contact-angle heterogeneity, further reshapes leakage pathways when integrated across molecular to aquifer scales.

Field studies and numerical models have demonstrated that small-scale structural features and variations in capillary properties can redirect plume pathways, alter trapping efficiency, and change leakage outcomes.^16,17,19^ Capillary heterogeneity has been recognized as a critical control on gas migration.^16,20–26^ However, field studies have shown that neglecting this factor may introduce systematic bias into gas plume migration predictions.^20–22,26,27^ Beyond porosity- and permeability-based scaling of capillary pressure, wettability represents an underexplored dimension of heterogeneity, with equally important consequences for plume behavior.

Conventional capillary pressure scaling in CH_4_ leakage studies and in related problems (e.g., CO_2_ and H_2_ storage) typically relies on spatial variation in porosity and permeability while holding interfacial properties fixed.^16,28–33^ This approach overlooks the fact that wettability depends on mineralogy and water chemistry (e.g., salinity), which changes local capillary entry pressures and, in turn, migration pathways but is rarely included in leakage modeling.

Direct measurements of contact angle (e.g., sessile/pendant drop) are valuable but face well-known challenges, such as surface contamination, unwanted roughness, and damage due to smoothing during core preparation. As a result, the measured contact angle may not truly represent the natural surface conditions. These challenges are particularly acute in unconsolidated shallow aquifers.^34,35^ Molecular dynamics (MD) simulation provides a complementary approach by resolving atomic-scale interactions at mineral–fluid–fluid interfaces. Prior studies show good agreement between MD-computed and laboratory-measured contact angles across a range of rock–fluid systems.^36–40^ In this way, MD simulation can supply facies-specific wettability inputs that can be transferred to continuum-scale models through the Leverett *J*-function, thereby making an explicit link between nanoscale surface physics and continuum-scale multiphase flow.

Here, we explore the link between microscale wettability and continuum-scale leakage behavior by integrating contact angles, computed from MD simulations as functions of mineral type and salinity, into continuum-scale multiphase flow simulations of CH_4_ leakage along a shallow aquifer wellbore. This integration is achieved using a contact-angle-informed Leverett *J*-function and evaluates how explicitly representing heterogeneous wettability influences CH_4_ migration, trapping, and leakage. We further identify the conditions under which this representation matters most by varying salinity, permeability contrast, leakage rate, and facies proportions within geologically plausible architectures.

## 2 Materials and Methods

Integrating MD simulation-computed, mineral- and salinity-dependent contact angles with continuum-scale multiphase flow simulations were performed in two stages. First, MD simulations provided facies-specific contact angles under varying salinity conditions. Second, the computed contact angles were incorporated into continuum-scale models to refine the capillary pressure scaling. The resulting simulations were used to quantify CH_4_ leakage to the atmosphere and to identify the conditions where quantifying contact-angle variability is most critical for accurate predictions. Computational costs for both MD simulations and continuum-scale simulations are detailed in

All MD simulations were performed with the LAMMPS package^41^ with MPI parallelization and GPU acceleration.^42–47^ Complete details of the force fields, equilibration protocols, and analysis procedures along with comparison with prior lab results are provided in . Although shallow aquifers contain multiple mineral phases, previous studies indicated that small-scale mineralogical heterogeneity exerts limited influence on solute transport, with plume migration and spreading governed primarily by larger-scale hydrogeologic structure.^25^ Based on the evidence, sediment variability was simplified into two principal monomineralic facies: coarse grain (CG) and fine grain (FG) facies (i.e., bodies of sediment).^48^ Quartz was selected as the representative mineral for the CG facies, while kaolinite-coated surfaces represented the FG facies.

Quartz exists in various polymorphic forms, each characterized by a unique atomic arrangement, depending on the pressure and temperature conditions of the porous medium.^49^ Among these, α-quartz with a (0001) surface is selected due to its stability under the target simulation conditions.^49,50^ Since α-quartz crystallizes in a hexagonal structure and the simulation box requires a rectangular geometry, modifications to the crystal lattice are necessary. To achieve this, the α-quartz unit cell^51^ is duplicated along the *x* and *y* directions and then trimmed along specific crystallographic planes to ensure two-dimensional periodicity and neutral charge of the substrate. An orthogonal unit cell is then constructed and replicated along the *x*, *y*, and *z* axes, maintaining atomic stoichiometry and bond connectivity throughout the structure.^52^ A Q2-cut is extracted from the generated α-quartz surface and fully hydroxylated to represent realistic aqueous conditions. For Q2, the silicon atom is bonded to two bridging oxygen atoms and two hydroxyl (OH) groups. This is a less stable, highly reactive silanol group found on the surface of silica, with the structure (≡Si–O−)_2_Si­(OH)_2_. In contrast, kaolinite (Al_2_Si_2_O_5_(OH)_4_) is a nonswelling, 1:1 type clay mineral composed of one tetrahedral silica (T) sheet and one octahedral alumina (O) sheet.^53^ Unlike smectites, kaolinite has negligible layer charge, and its interlayers are stabilized by hydrogen bonding between hydroxyl groups on the alumina sheet and basal oxygen atoms on the silica sheet, forming rigid TO–TO stacking with minimal interlayer expansion. The kaolinite unit cell is first extended in the *x* and *y* directions and is then replicated three times along the *z*-axis to generate the simulation domain.

Water–CH_4_ contact angles on these minerals were estimated using established MD simulation protocols that have been shown to reliably reproduce laboratory-scale measurements.^38–40^ In this study, a semicylindrical water droplet was placed on the mineral substrate in the presence of CH_4_ and equilibrated to steady geometry (Supporting Information, Figure S1). In the simulation box, periodic boundary conditions are applied in the *x*- and *y-*directions to simulate an infinite system, ensuring that when a particle exits one boundary, it seamlessly re-enters from the opposite side. In contrast, nonperiodic boundary conditions are enforced in the *z*-direction, where atoms may be reflected, absorbed, or constrained within the simulation box. To minimize boundary effects, a reflective wall is placed at the top of the simulation box, and the box height was selected to be sufficiently large to prevent any influence of the walls on the contact-angle measurements. The size of the simulation box is about 120 nm in the *z*-direction, including the substrate. The contact angle was measured at the three-phase boundary between the water droplet, mineral surface, and CH_4_ phase to be used in continuum-scale simulations.

The concept of the reservoir elementary volume (REV) is fundamental to continuum-scale reservoir simulations, as it defines the minimum scale at which properties such as wettability and capillary pressure become representative and suitable for upscaling.^54^ In this study, the REV assumption is explicitly addressed by employing MD simulations to quantify contact angles that are consistent with laboratory-scale measurements. The close agreement between the MD-derived contact angles and published experimental data demonstrates that the simulations reliably capture the wettability behavior from the pore scale to the laboratory scale. Because capillary pressure functions used in continuum models inherently rely on REV-averaged wettability parameters, the MD-derived contact angles fall within the appropriate representative scale for upscaling and can be directly incorporated into continuum-scale multiphase flow simulations as physically meaningful REV-consistent inputs.

Continuum-scale simulations were conducted using the GEM module of the Computer Modeling Group (CMG, Version 2024.30). The range of the properties used in continuum-scale simulations is based on prior field studies and numerical simulations in CH_4_ leakage in unconfined shallow aquifers.^17,24^ The model domain was divided into three sections representing an unconfined shallow aquifer with an overlying vadose zone and atmosphere (Supporting Information, Section S3). Initial saturations were specified as 100% water in the aquifer, 40% water and 60% air in the vadose zone, and 100% air in the atmosphere. The CH_4_ leakage was introduced as a point source in the aquifer to simulate upward and lateral migration. Air was modeled as a pseudopure component, assumed insoluble under shallow subsurface conditions.^55^ Fluid properties and phase behavior were calibrated using CMG’s Winprop module (Supporting Information, Section S3.1). Section-specific relative permeability and capillary pressure functions were assigned to represent the multiphase flow behavior and potential leakage to the atmosphere.

Although capillary pressure curves are typically assigned at the facies scale,^56^ small-scale variations in mineral composition, grain coatings, cementation, and clay content occur within a single facies. Capillary heterogeneity associated with this subfacies mineral heterogeneity can be explicitly incorporated using our approach.

Capillary pressure scaling for each grid block incorporated wettability variability via a contact-angle-informed (CA) Leverett *J*-function:^57^

$$\frac{P_{c,\mathrm{grid}}}{P_{c,\mathrm{ref}}}=\frac{\cos(\theta_{\mathrm{grid}})}{\cos(\theta_{\mathrm{ref}})}\sqrt{\frac{k_{\mathrm{ref}}\phi_{\mathrm{grid}}}{k_{\mathrm{grid}}\phi_{\mathrm{ref}}}}$$ (1)

where subscripts ref and grid refer to reference conditions and model grid block values. *k*, ϕ, θ, and *P*_*C*_ represent absolute permeability, porosity, contact angle, and capillary pressure, respectively.

The first term ( $\frac{\cos(\theta_{\mathrm{grid}})}{\cos(\theta_{\mathrm{ref}})}$ ) accounts for wettability variability between facies, while the second term ( $\sqrt{\frac{k_{\mathrm{ref}}\phi_{\mathrm{grid}}}{k_{\mathrm{grid}}\phi_{\mathrm{ref}}}}$ ) accounts for scaling of pore-size correlation length. In the no-contact-angle (NCA) scenario, wettability is assumed constant, so the first term equals unity and the scaling reduces to the conventional porosity–permeability formulation used in most gas transport studies.

Each simulation began with a 30 day preleakage phase, during which injection and production wells at model boundaries operated at about 0.6 m^3^/day to establish steady-state groundwater flow. The CH_4_ leakage was then introduced at a constant rate of 0.14 rm^3^/day for 60 days, and simulations were extended up to 100 days to ensure stabilization of the leakage pathways.

The spatial distribution of CG and FG facies within both the aquifer and vadose zone was modeled using a transition-probability-based approach implemented in T-PROGS^58^ (see Supporting Information, Section S3.2). This method generated 25 stochastic realizations of the facies architecture, each representing a distinct spatial configuration of CG and FG facies while maintaining identical volumetric proportions. The realizations differ in the geometry, spatial arrangement, and connectivity of the facies, thereby capturing geological uncertainty and allowing the assessment of stochastic variability in model outcomes.

To represent different hydrogeochemical conditions, both MD and continuum-scale simulations were conducted for zero-salinity (0 ppm NaCl) and saline (12,000 ppm NaCl) groundwaters. The freshwater case represents inland aquifers recharged primarily by precipitation, while the saline case represents coastal aquifers, evaporative basins, or regions influenced by agricultural recharge.^59–62^

For the base scenario, the permeability of CG facies, CH_4_ leakage rate, and volumetric proportion of CG facies were set to 5,000 mD, 0.14 m^3^/day, and 40%, respectively. From this baseline, additional scenarios were developed by systematically varying key parameters: (i) increasing CG permeability from 5,000 to 20,000 mD while raising the log-scale variance from 0.3 to 1.0; (ii) increasing CH_4_ leakage rate and total leakage volume by a factor of 2.5; (iii) increasing salinity from 0 to 12,000 ppm to assess the influence of groundwater chemistry; and (iv) increasing the volumetric proportion of CG facies from 40 to 60% to examine the effects of sedimentary variability and connectivity. The upper limit of 60% CG facies was selected because, in two-dimensional systems, the connectivity threshold occurs near 50%. The resulting CH_4_ distributions across mobile, trapped, and dissolved phases were then compared between contact-angle-included (CA) and NCA scenarios to evaluate the influence of contact angle on leakage predictions.

## 3 Results and Discussion

### 3.1 Contact-Angle Estimation

Estimated contact angles from MD simulations are listed in Figure 1. To confirm that simulation time was sufficient, the normalized vertical position of the water droplet center of mass was tracked for each mineral at two salinity levels (0 and 12,000 ppm NaCl). As shown in Supporting Information, Figure S2, all cases reached a plateau, indicating that equilibrium was achieved (6–10 and 2 ns for quartz and kaolinite, respectively). The MD-computed contact angles fall within the range of previously reported laboratory measurements for quartz (Supporting Information, Figure S3), supporting the reliability of the approach. Variability among experimental values likely reflects differences in sample preparation, surface conditions, measurement techniques, and operator handling. The consistency between our MD results and this experimental range further supports the validity of the modeling approach; however, the uncertainty observed in laboratory measurements should also be acknowledged and incorporated into the analysis.

**Figure 1:**
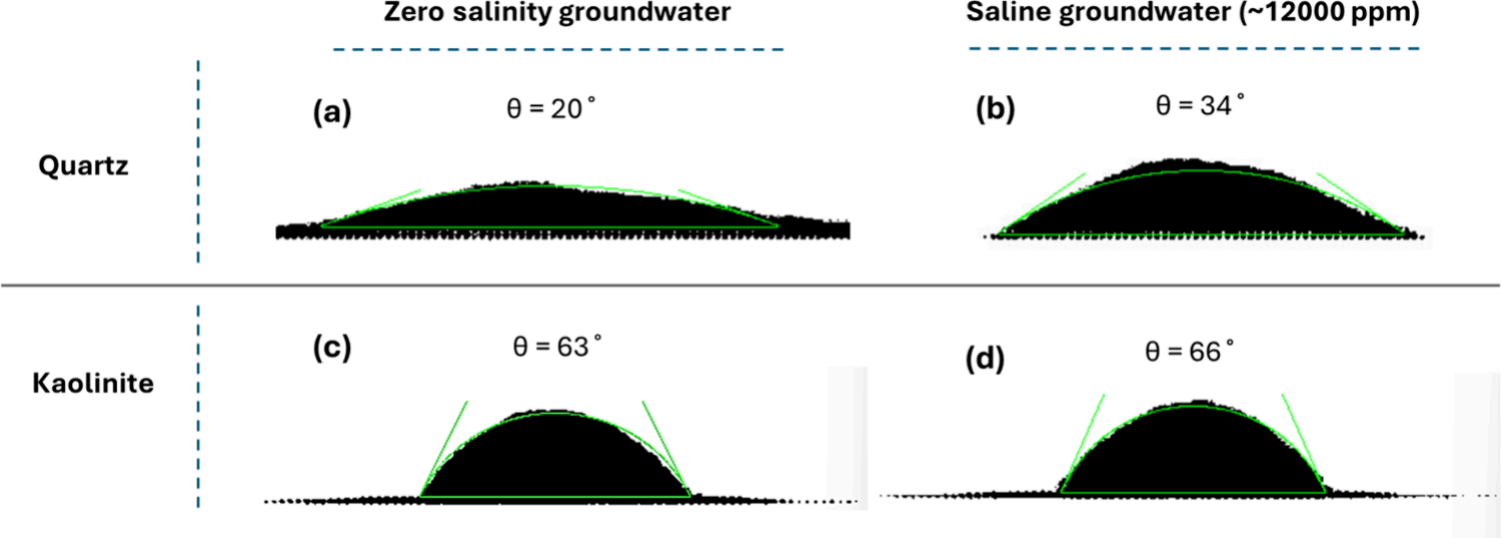
Estimated contact angle using molecular dynamics simulation based on the time-averaged droplet shape for the last 2 ns during the production phase for the water–CH_4_–substrate system: (a) Zero salinity groundwater–CH_4_–quartz, (b) saline groundwater–CH_4_–quartz, (c) zero salinity groundwater–CH_4_–kaolinite, and (d) saline groundwater–CH_4_–kaolinite. The light green lines indicate the boundaries of the water droplet used for contact-angle determination.

Quartz is consistently more water-wet than kaolinite. Increasing salinity reduces water wetness on both minerals, raising the contact angle (θ) by about 14° on quartz (from about 20° to about 34°) and by about 3° on kaolinite (from about 63° to about 66°). The stronger salinity response on quartz is physically reasonable^63–68^ as dissolved salts screen electrostatic charges at the mineral–water interface, weakening water’s adhesion to the surface and enhancing gas wettability. The uncertainty in contact angle was estimated for both minerals under zero salinity and saline conditions using 10 frames from the final 1 ns of the MD simulations. For quartz, the standard deviation of the contact angle is approximately 1.7° in zero salinity groundwater and 2.6° in saline water, whereas for kaolinite, it is about 3.7 and 4.0°, respectively.

### 3.2 Continuum Scale Simulations

After completing all simulations, we first examined how the average CH_4_ (%) distribution converged as the number of realizations increased. This convergence analysis, conducted for the base scenario after 190 days of simulation, considered variations across different reservoir sections and fluid phases. The objective was to verify the statistical stability and representativeness of the simulation results, ensuring that the observed CH_4_ migration and trapping behaviors were not influenced by random variability in mineral distributions (Supporting Information, Section S4.1). Once convergence was confirmed, plume propagation was compared between the CA and NCA scenarios.

As shown in Figure 2, incorporating contact-angle variability into capillary pressure scaling (CA) fundamentally reshapes the spatial distribution of the capillary entry pressure in the reservoir. By reducing sharp contrasts between high- and low-entry-pressure regions, this approach produces a smoother and more spatially distributed capillary heterogeneity pattern compared with conventional scaling without contact-angle variability (NCA). In the NCA case, clay-coated regions retain high entry pressures and act as capillary barriers, forcing CH_4_ to migrate through a limited number of preferential pathways and resulting in sharp plume fronts. When contact-angle variability is included, local reductions in cos­(θ) lower entry pressures in portions of these regions, enabling CH_4_ to invade previously inaccessible pore spaces and activating additional flow pathways. This redistribution of entry pressure alters the plume morphology, yielding a smoother plume front and modifying lateral plume spreading, as illustrated in Figure 2b.

**Figure 2:**
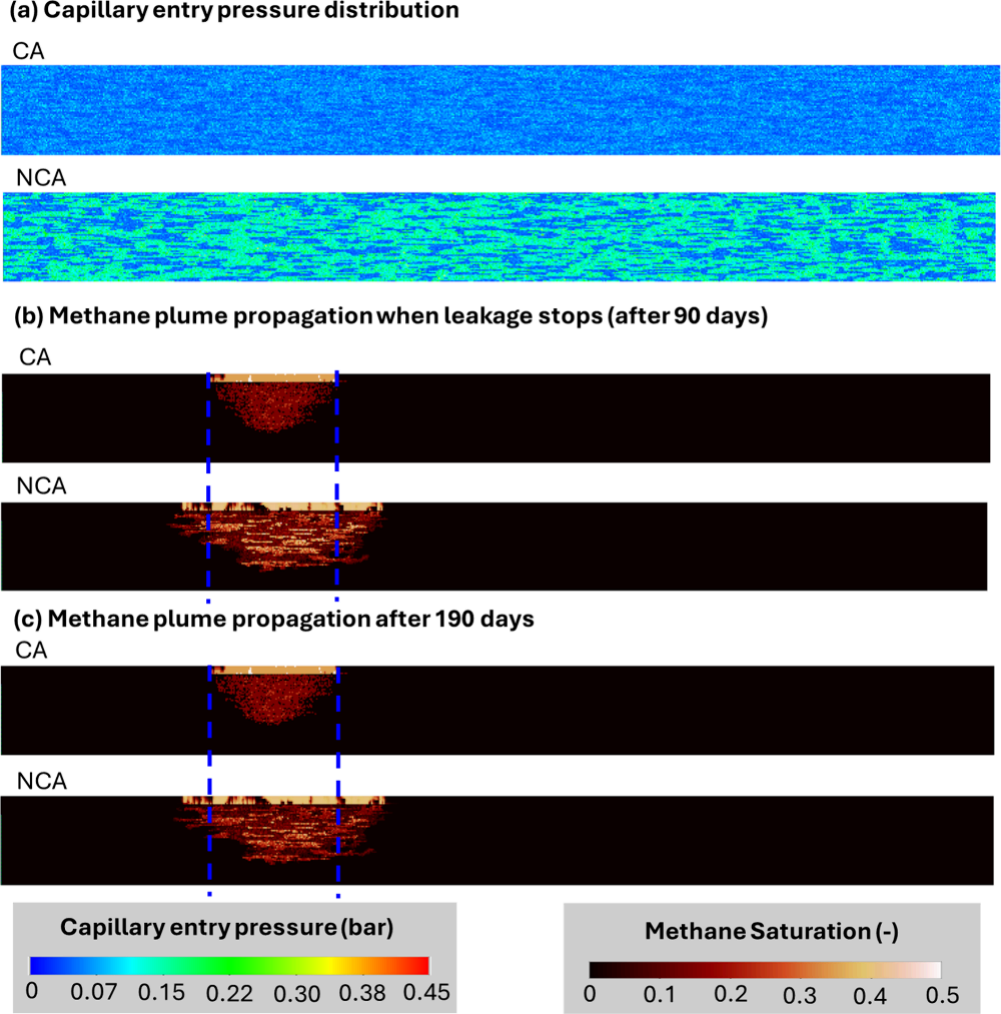
Comparison of the effect of contact-angle inclusion on heterogeneity in capillary entry pressure and leaked CH_4_ plume propagation in a representative realization of the base scenario: (a) Capillary entry pressure distribution, (b) CH_4_ plume propagation when leakage stops (after 90 days), and (c) CH_4_ plume propagation after 190 days. CA and NCA stand for contact angle included and excluded scenarios, respectively.

A comparison between Figure 2b,c further shows that once the leakage source is shut off, lateral CH_4_ migration becomes minimal. Plume evolution is then dominated by upward migration, driven by buoyancy or by immobilization through capillary trapping. At this stage, capillary forces shaped by heterogeneous entry pressures, together with gravitational forces arising from the density contrast between CH_4_ and groundwater, outweigh viscous forces that would otherwise promote horizontal spreading. Overall, these results demonstrate that contact angle variability weakens effective capillary barriers, redistributes methane flux, and fundamentally alters migration pathways, with important implications for methane leakage behavior.

Animations of CH_4_ plume migration through the aquifer and vadose zone for a representative scenario are provided in Video S1. Results show that incorporating contact angle variability generates additional flow pathways that reduce the sharp plume fronts typically observed when only conventional capillary heterogeneity is considered. CA also leads to higher leakage to the atmosphere compared to NCA. Moreover, the animations illustrate how the mobile CH_4_ phase in the aquifer gradually breaks into disconnected clusters once the injection stops, indicating the loss of hydraulic connectivity. These isolated gas clusters become increasingly immobilized over time due to capillary trapping, which prevents further upward migration and contributes to the long-term stabilization of CH_4_ in the subsurface.

As demonstrated, contact angles computed from MD simulations inherently contain some degree of uncertainty. Comparison with experimental measurements indicates that the MD-derived contact angles fall within the range of laboratory results; however, certain unaccounted factors may still influence the wettability behavior. To account for these potential effects, a sensitivity analysis was conducted by varying the contact angle of each facies by ±10°. To capture the range of possible outcomes, continuum-scale simulations were conducted for two extreme scenarios representing maximum and minimum contrasts in facies-specific contact angles. These scenarios were used to assess the influence of contact-angle uncertainty on CH_4_ leakage predictions. Further details are provided in Supporting Information, Section S4.2.

Figure 3 illustrates the uncertainty of temporal evolution of CH_4_ partitioning across the aquifer, vadose zone, and atmosphere for 25 stochastic realizations, comparing the NCA and CA cases. These variations in facies configuration led to differences in plume migration and trapping behavior, which are reflected in the variability shown in the figure. Within the aquifer and vadose zones, CH_4_ was categorized into mobile, snap-off trapped, and dissolved phases. In the simulator, the dissolved CH_4_ is computed based on the partitioning of CH_4_ into the aqueous phase using Henry’s law, while snap-off trapped methane is quantified using the Land trapping model (see Supporting Information, Section S3, for details). The mobile CH_4_ in each grid block is then obtained by subtracting the dissolved and residually trapped CH_4_ from the total CH_4_ present. As CH_4_ quantities in the vadose zone and their associated variability were relatively small, only the total value is reported here (see Supporting Information, Section S4.3 for details on trapped CH_4_ in the vadose zone). In the atmosphere, only total CH_4_ is presented, as CH_4_ exists solely in its gaseous form in this region. In the figure, each row corresponds to a specific sensitivity scenario on the base scenario such as increased permeability, elevated salinity, higher CH_4_ leakage, or increased volume proportion of CG facies, while each column shows the temporal distribution of CH_4_ across the different sections or phases. The resulting CH_4_ percentages represent the fraction of CH_4_ moles in each phase or section relative to the total leaked CH_4_ in each simulation. Variability was quantified as the standard deviation from the mean across the 25 realizations, reflecting the influence of geological heterogeneity on plume propagation and phase partitioning. In the plots, solid lines indicate mean values, shaded regions denote one standard deviation above and below the mean, and gray shaded areas highlight regions of overlap between the CA and NCA cases, illustrating where their results converge.

**Figure 3:**
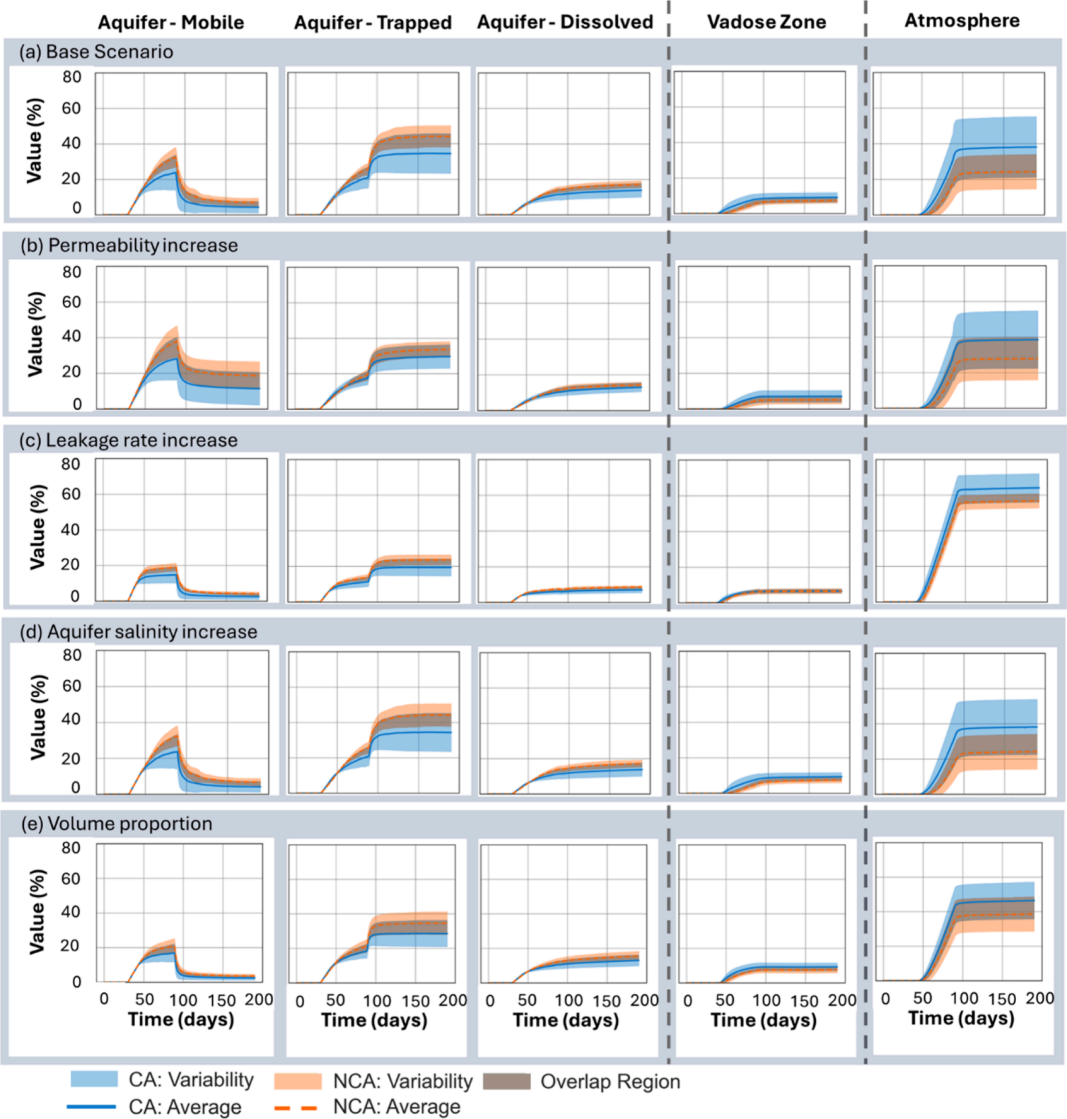
Variability of the volume percentage of leaked CH_4_ across 25 stochastic realizations of mineral heterogeneity. Simulations were performed in the aquifer, vadose zone, and atmosphere over 190 days, comparing cases with and without contact-angle incorporation. Scenarios include (a) the base scenario, (b) permeability increase (4 × base), (c) leakage rate increase (2.5 × base), (d) aquifer salinity increase (changed from 0 to ∼12,000 ppm), and (e) volume proportion increase (coarse-grain (CG) volume proportion changed from 40 to 60%). In the aquifer, results are shown for mobile CH_4_ gas, snap-off trapped CH_4_, and dissolved CH_4_; in the vadose zone and atmosphere, only the total CH_4_ gas is shown. Abbreviations: CA = contact angle included in capillary pressure scaling; NCA = no contact angle included.

Within the aquifer, mobile CH_4_ concentration increases during the leakage phase and then declines after leakage stops as CH_4_ becomes trapped, is dissolved, or migrates upward. Among the phases, dissolved CH_4_ shows the least variability (about 10%). Incorporating contact-angle heterogeneity reduces aquifer CH_4_ by up to 10% in both mobile and trapped phases, while atmospheric leakage increases by as much as 20%. In contrast, the vadose zone exhibits only a minor increase in CH_4_, with negligible differences between the CA and NCA averages.

At higher leakage rates, differences between CA and NCA scenarios diminish and variability across realizations decreases. The reduction arises because viscous-dominated flow suppresses capillary effects, leading to enhanced CH_4_ migration into the atmosphere regardless of wettability assumptions.

While Figure 3 illustrates variability using standard deviations symmetrically around the mean, the approach may obscure the full range of outcomes across realizations. To more explicitly capture the spread in CH_4_ distribution, we evaluated the results after 190 days using box plots (Figure 4), which provide a clearer representation of variability among scenarios in each section. Each box represents the interquartile range (25th–75th percentile), with the central line indicating the median. Whiskers show the spread of nonoutlier data, and individual markers denote outliers. This statistical representation highlights both the central tendency and the variability across scenarios with and without contact-angle (CA versus NCA) effects.

**Figure 4:**
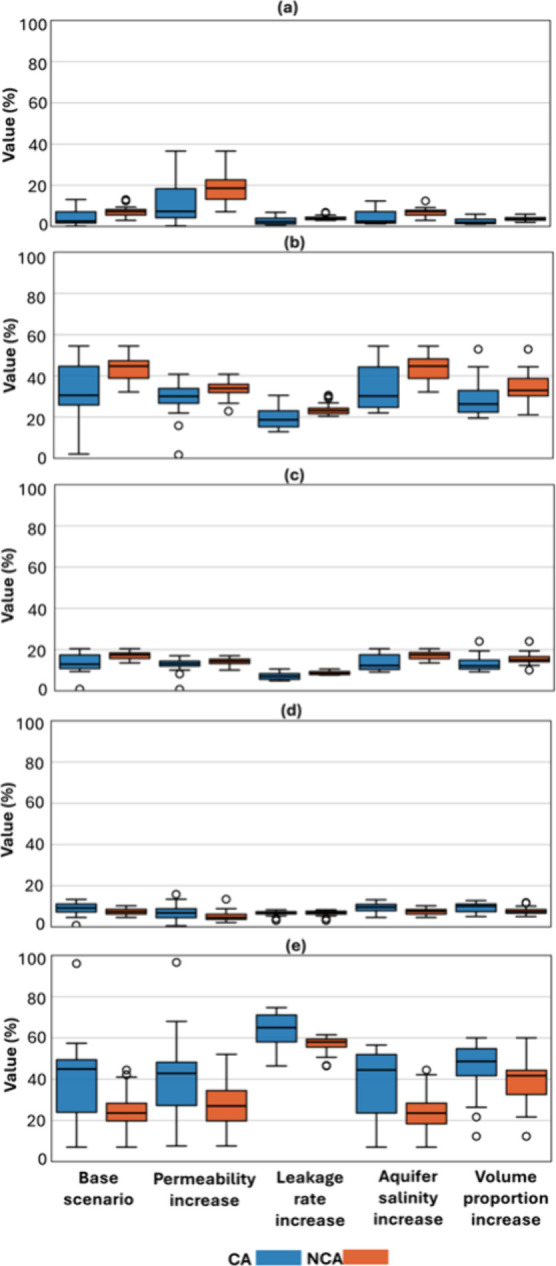
Volume percentage of leaked CH_4_ across the aquifer, vadose zone, and atmosphere after 190 days, when the CH_4_ plume stabilized in different scenarios: Base scenario, permeability increase, leakage rate increase, and volume proportion increase. Results are shown for scenarios with and without contact-angle incorporation. (a) Mobile CH_4_ gas in aquifer, (b) snap-off trapped CH_4_ in aquifer, (c) dissolved CH_4_ in aquifer; (d) total CH_4_ gas in the vadose zone; (e) total CH_4_ gas in the atmosphere. CA = contact angle included in capillary pressure scaling; NCA = contact angle not included; and CG = coarse-grain rock type.

Comparison of interquartile ranges between the CA and NCA cases indicates that incorporating the contact angle enhances the variability associated with mineral heterogeneity, thereby increasing the influence of stochastic realizations. Among all scenarios, the largest variability in mobile CH_4_ is observed under a permeability increase, where the interquartile range is the widest and the median difference between CA and NCA reaches nearly 10%. In contrast, scenarios involving an increased leakage rate or volume proportion exhibit the smallest differences, both in variability and median values.

For snap-off trapped CH_4_, the interquartile range generally increases when the contact angle is included, except in the permeability increase case. Median values exhibit the largest CA–NCA difference (about 20%) in the base case and in the salinity increase scenario, although the absolute values differ. This suggests that aquifer salinity, which can vary with environmental conditions, influences CH_4_ partitioning across phases. Notably, despite the variations, the difference between CA and NCA remains consistent in the trapped phase, indicating that contact-angle effects must be incorporated for accurate quantification of CH_4_ trapping. Dissolved CH_4_ follows a trend similar to that of trapped CH_4_.

In the vadose zone, CA scenarios consistently yield slightly higher mobile CH_4_ fractions compared with NCA, with median differences below 3%. The exception is the leakage rate increase scenario, where both median and variability are nearly identical. CH_4_ percentages in this section range between 9–12% for CA, reflecting the absence of capillary trapping due to lose sediments and the transitional role of the vadose zone between aquifer and atmosphere.

Atmospheric leakage is also the other critical factor.^6,10^ As expected, scenarios with increased leakage rate and volume result in the greatest CH_4_ release to the atmosphere, with median values of approximately 75% for CA and 70% for NCA. These findings highlight the importance of incorporating contact angle variability into modeling efforts, as it alters CH_4_ partitioning among phases and directly affects the magnitude of emissions reaching the atmosphere.

## 4 Limitations, Implications, and Future Research

The modeling framework developed in this study is intended to examine the relative influence of heterogeneous wettability and capillary heterogeneity on CH_4_ migration and trapping at the continuum scale. As such, several assumptions and limitations should be recognized with the results. First, the simulations are based on continuum-scale multiphase flow governed by Darcy’s law and assume local capillary equilibrium within each grid block. Pore-scale processes such as film flow, ganglion dynamics, and dynamic contact angle behavior^69^ are not explicitly resolved. Wettability is represented by using static contact angles derived from MD simulations. Although these values are consistent with laboratory-scale measurements, potential time-dependent wettability alterations driven by geochemical reactions or biological activity^70–72^ are not considered. Second, the capillary pressure–saturation and relative permeability–saturation relationships are defined using constitutive models that rely on empirical parametrizations.^29^ Relative permeability hysteresis is included to capture drainage and imbibition behavior; however, owing to the limited availability of experimental hysteresis data for CH_4_–water systems under relevant conditions, an established empirical model is employed. Consequently, estimates of residual trapping and snap-off should be interpreted as indicative of general trends rather than precise quantitative predictions. Third, mass transfer between gas and aqueous phases is treated under equilibrium assumptions, while reactive transport, microbial CH_4_ consumption, and geomechanical deformation are neglected. Although these processes may be important in specific field settings, they are excluded here to isolate the effects of wettability-driven capillary heterogeneity. Within these constraints, the results provide mechanistic insight into how heterogeneous wettability can modify methane mobility, influence capillary trapping efficiency, and alter leakage pathways. The framework is therefore best viewed as a sensitivity-based and process-oriented tool that complements laboratory and field observations and informs future more comprehensive modeling efforts.

Our results provide a proof of concept for the previously underexplored role of heterogeneous wettability in generating capillary heterogeneity, extending earlier field-calibrated and simulation-based studies that demonstrated the influence of capillary heterogeneity on CH_4_ plume geometry and fate in shallow aquifers.^16,17,73^ By isolating wettability as a distinct mechanism, this work enhances the fundamental understanding of methane migration and trapping processes and helps guide future experimental, field, and monitoring investigations aimed at directly evaluating wettability-driven effects. Meanwhile, simplifying or ignoring capillary heterogeneity leads to overestimated atmospheric CH_4_ and underestimated mobile CH_4_ in the aquifer.^15^ We demonstrate that heterogeneous wettability, expressed through spatially variable water–CH_4_ contact angles as a function of mineralogy and salinity, adds an independent and quantifiable effect by directly modifying local capillary entry pressures, even when permeability and porosity are fixed. Incorporating contact-angle variability (CA) changes partitioning: aquifer mobile and snap-off trapped CH_4_ decrease by up to 10%, while atmospheric leakage increases by up to 20%. The CA–NCA difference decreases at high leakage rates, consistent with a transition toward viscous-dominated flow.

Our findings have important practical implications for leakage risk assessment. Conventional models that assume constant wettability (NCA) underestimate atmospheric leakage in settings where mineralogy and groundwater salinity covary, such as quartz-rich coastal or evaporite aquifers.^16^ This bias arises because salinity increases the contact angle on quartz much more than on kaolinite, lowering capillary entry pressures in quartz-dominated saline systems and promoting earlier gas breakthrough and higher leakage flux. Conversely, decreasing groundwater salinity can reduce the contact angle and raise capillary entry pressures, thus enhancing gas trapping and limiting leakage. To quantify these dynamics, a contact-angle-informed Leverett *J*-function, eq 1, should replace conventional scaling that accounts only for porosity and permeability, particularly in mixed-facies aquifers with sharp mineralogical contrasts or evolving salinity. This approach complements the findings of Ershadnia et al. (2021),^16^ which highlighted the dominant role of a meter-scale facies architecture in controlling plume migration. They demonstrated that heterogeneous wettability acts on the same scales and must therefore be jointly characterized, mapped, and modeled to improve predictions of subsurface gas transport and leakage risk.

Recent advances in micro-CT imaging have enabled the direct extraction of contact-angle fields from pore-scale images, demonstrating that wettability is inherently spatially heterogeneous rather than taking on a single uniform value.^74^ Building on this evidence, a practical approach for applying CT results at the field scale is to use CT data as a facies-level calibrator. In this workflow, a limited number of representative core plugs from each facies can be imaged, and their contact-angle distributions can be computed. The resulting statistics can then be mapped as a facies-conditioned contact-angle field throughout the reservoir model. Our contact-angle–informed *J*-function is designed to incorporate such fields directly, enabling propagation of pore-scale heterogeneity into continuum-scale flow simulations. Where CT data are unavailable, MD simulation-computed contact angles serve as a physically grounded alternative that can later be refined as even limited CT measurements become available. This hybrid strategy circumvents the challenges of direct CT-to-field upscaling, ties wettability estimates to measured rock textures, and naturally facilitates uncertainty quantification through ensemble sampling alongside variations in the permeability contrast, leakage rates, and salinity.

In this study, we focused on clean quartz and kaolinite surfaces and assumed static contact angles; however, natural sediments often exhibit additional complexities such as surface roughness (asperities), organic coatings, iron oxide precipitates, and contact-angle hysteresis (advancing vs receding angles), all of which can significantly influence wettability and plume migration. Future experimental and modeling efforts should address the effect of these factors to better represent realistic leakage in unconfined shallow aquifers. Moreover, salinity was held constant in our simulations, whereas in nature, groundwater chemistry evolves over time due to changing boundary conditions, which in turn can alter the contact-angle and capillary behavior. Continuum-scale measurements of contact-angle variability and leaked CH_4_ plume propagation across space and time are therefore crucial to validate and refine predictive models and to capture the dynamics of CH_4_ leakage. Such data, combined with improved models, will enhance the accuracy of risk assessments for groundwater quality and fugitive CH_4_ emissions.

The implications of our findings extend beyond shallow aquifers. Heterogeneous wettability remains a critical unknown in deep geologic systems, including CO_2_ and H_2_ storage or toxic chemicals such as NAPL, where high mobility ratios make capillary heterogeneity, a key factor controlling plume propagation and contributing to short-term trapping.^19–21,24,28,29,75,76^ Moreover, injected streams may contain or interact with residual CH_4_ due to coinjection, legacy hydrocarbons, or impurity carryover from capture and transport processes.^77–80^ As a result, interactions involving CH_4_ should also be considered when evaluating flow and trapping behavior at larger spatial scales, particularly given prior field-calibrated evidence that capillary heterogeneity exerts first-order control on CH_4_ plume migration and leakage.^16^ Addressing such knowledge gaps by integrating laboratory and field with multiscale modeling studies will improve predictive capabilities for both shallow and deep subsurface fluid migration and support the development of safer and more effective energy and climate mitigation technologies.

## Supplementary Material





## Data Availability

The simulation samples from both molecular dynamics and continuum-scale models generated in this study are publicly available on Zenodo [10.5281/zenodo.17459998], enabling access and reproducibility of the results.

## Notes

**A**dditional details on computational costs, molecular dynamics simulation setup, force-field parameters, contact angle analysis procedures, conceptual modeling of methane leakage, fluid property calibration, stochastic sediment realizations, and extended results on methane distribution and uncertainty in contact angle estimates (PDF)
Video S1: Comparison of methane plume propagation in the presence and absence of heterogeneous wettability (MP4)

## Abbreviation

CA, NCA: Contact angle included, excluded scenarios
FG, CG: Fine, coarse grain
MD: Molecular dynamics
**Chemical formula**
CH_4_: Methane
CO_2_: Carbon dioxide
H_2_: Hydrogen
**Parameter**
*k*: Permeability in mD
ϕ: Porosity in fraction
*P*_*c*_: Capillary pressure
*j*(*S*_*w*_): *J*-function as a function of water saturation
σ: Interfacial tension
θ: Contact angle
**Units**
Bar: Metric unit of pressure
nm: Nanometer
mD: Milli-Darcy
g/mol: Gram per mole
K: Kelvin
ppm: Part per million
rm^3^/day: Cubic meter per day at aquifer condition
