# Supplementary material for: Heterogeneous Wettability Alters Methane Migration and Leakage in Shallow Aquifers
Source: Environ Sci Technol. 2026 Mar 27;60(13):9991–10000. doi: 10.1021/acs.est.5c15451 (PMC13063803; doi:10.1021/acs.est.5c15451)
Supplement: Supplementary file 1 [file es5c15451_si_001.pdf]

Supporting Information

Heterogeneous Wettability Alters Methane Migration and Leakage in Shallow Aquifers

Sabber Khandoozi[a,*], Siddharth Gautam[b], Craig Dietsch [a], Muhammad Sahimi[c], David Cole[b], Reza Soltanian[a,d,**]

[a]Department of Geosciences, University of Cincinnati, Cincinnati, OH, 45221, USA

[b]School of Earth Sciences, The Ohio State University, Columbus, OH, 43210, USA

[c]Mork Family Department of Chemical Engineering and Materials Science, University of Southern California, CA, 90089-1211, USA

[d]Department of Environmental Engineering, University of Cincinnati, Cincinnati, OH, 45221, USA

Corresponding authors: [*]khandosr@uc.edu

[**]soltanma@uc.edu

Contents

S1 Computational costs 2
S2 Molecular dynamics simulation 2
S2.1 Results of molecular dynamics simulation 5
S2.2 Comparison of molecular dynamics simulation results with lab results....6
S3 Conceptual model 8
S3.1 Fluid properties....12
S3.2 Sediments properties 12
S4 Results of continuum-scale simulations....14
S4.1 Quantitative distribution of methane....14
S4.2 Uncertainty in contact angle....14
S4.3 Methane in vadose zone 17
References 18

S1 Computational costs

All molecular dynamics (MD) simulations were conducted on the Pitzer cluster at the Ohio Supercomputer Center. Simulations utilized 8 compute nodes, each configured with 2–8 MPI tasks and equipped with 2 GPUs per node, which provided an optimal balance between computational efficiency and communication overhead. Under this configuration, each MD simulation required approximately 13 hours of wall-clock time.

Continuum-scale multiphase flow simulations were performed on a university-hosted Linux workstation equipped with 36 physical CPU cores and 256 GB of RAM. The simulations were executed in parallel to model 190 days of plume evolution, with an average computational cost of approximately 24 CPU-hours per simulation. A total of 350 simulations were conducted to explore variability associated with mineralogical distributions, salinity conditions, leakage scenarios and the randomization of volumetric mineral proportions.

S2 Molecular dynamics simulation

To set up the simulation, the substrate (~200×50×20 nm) is positioned at the base of the simulation box, serving as the foundation for the system. A semi-cylindrical water droplet is carefully placed at the center of the box, just above the substrate molecules (**Figure S1**). Unlike experimental investigations, where macroscopic droplets are commonly used, MD simulations typically rely on nanodroplets due to the high computational cost of simulating micro-scale droplets. However, nanodroplets exhibit distinct behaviors compared to larger experimental droplets, leading to scale-dependent effects in contact angle measurements. To mitigate such discrepancies, cylindrical droplets are used, which

help minimize the influence of three-phase contact line tension, thereby producing contact angle values more comparable to macroscopic droplets [1,2].

Surrounding the droplet, methane (CH_4) molecules filled the remaining space, creating a realistic representation of the fluid interactions at the solid-liquid-gas interface. The quartz and kaolinite substrate were treated as frozen except for their -OH bonds allowing the movement of the surface hydroxyl groups. Both the bonds of water molecule were constrained using the SHAKE algorithm. Visualization of atom trajectories was performed using the OVITO software package.

Interatomic interactions are modeled using the Lennard-Jones (LJ) and the Coulombic potential, both truncated at a cutoff distance of 1.2 nm. Long-range electrostatic interactions are computed using the particle–particle–particle–mesh (PPPM) method, ensuring accurate force calculations. To account for interactions between dissimilar atoms, the Lorentz–Berthelot mixing rule is applied. Atomic motion followed Newton's second law of motion, while intermolecular interactions are defined using well-established force fields: SPC/E for water [3], Clayff for quartz and kaolinite [4,5], OPLS-AA force fields for CH_4 [6] with a rigid body, and Joung-Cheatham for sodium chloride [7] (**Table S1**). The number of CH_4 molecules is determined using a generalized form of real gas equation of state (PV = ZnRT), where P represents pressure, V is volume, Z is the compressibility factor, n is the number of moles of CH_4, R is the universal gas constant, and T is temperature. In our simulations, as shallow aquifers are at almost atmospheric conditions, Z can be assumed to be 1.

The simulation began with energy minimization to remove excess potential energy from the initial configuration. Temperature is maintained constant using the Nosé–Hoover

thermostat [8], set with a damping factor of 20 picoseconds and a time step of 1 picosecond [9,10]. The total simulation time is 12 nanoseconds (ns), with data analysis performed on the final 2 nanoseconds to ensure equilibrium conditions are reached [9,10].

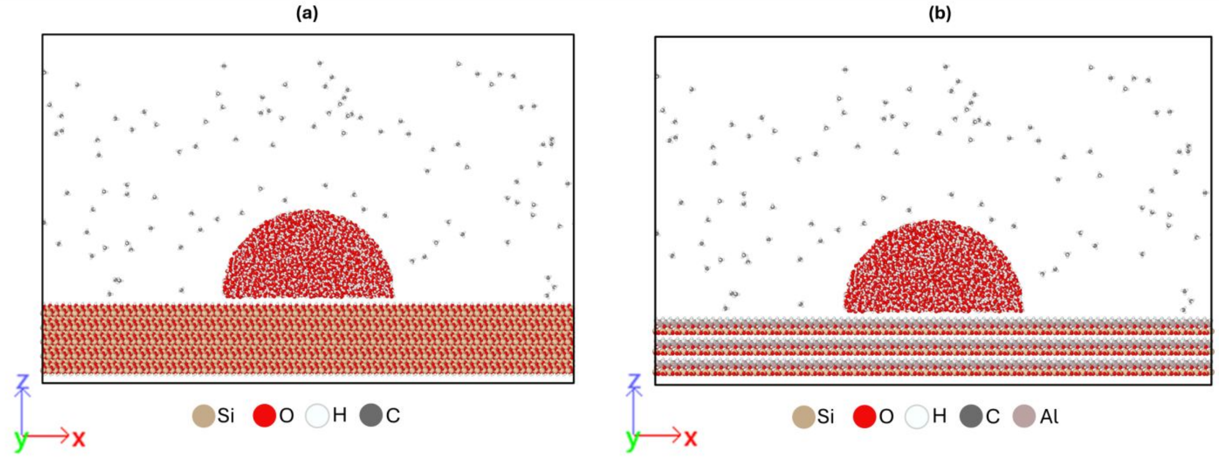

Figure S1. Initial molecular dynamics (MD) simulation configurations in the x–*z* plane for contact angle measurements: (a) quartz–water–CH_4, (b) kaolinite–water–CH_4.

Table S1. Total potential parameters for different forces: coulomb, van der Wals, bond and angle force fields. Forcefields are SPC/E for water [3], Clayff for quartz and kaolinite [4,5], OPLS-AA force fields for CH_4 [6], and Joung - Cheatham for sodium chloride [7].

Parameter	Potential parameters values						
	Coulomb	van der Waals		Bond		Angle	
	$\frac{e^2}{4\pi\varepsilon_0}\sum_{i\neq j}\frac{q_i q_j}{r_{ij}}$	$\sum_{i\neq j} 4\varepsilon_{ij}\left(\left(\frac{\sigma_{ij}}{r_{ij}}\right)^{12}-\left(\frac{\sigma_{ij}}{r_{ij}}\right)^{6}\right)$		$k_1(r_{ij}-r_0)^2$		$k_2(\theta_{ijk}-\theta_0)^2$	
	$q_{i\ or\ j}$ (eV)	$\varepsilon_{i\ or\ j}$ (kcal/mol)	$\sigma_{i\ or\ j}$ (Å)	k_1	r_0	k_2	θ_0
Water (H_2O)							
H	0.4328	0	0	-	-	-	-
O	-0.8476	0.15535	3.166	-	-	-	-
Methane (CH_4)							
C	-0.2400	0.06600	3.500	-	-	-	-
H	0.0600	0	0	-	-	-	-
α-Quartz (SiO2)							
$Si_{b/h}$	2.1000	1.840e-6	3.302	-	-	-	-
Si_m	1.0500	1.840e-6	3.302	-	-	-	-
O_b	-1.0500	0.15539	3.166	-	-	-	-
O_h	-0.9500	0.15539	3.166	-	-	-	-
H_h	0.0600	0	0	-	-	-	-
O_h-H	-	-	-	554.1349	1.0		
Si-O_h-H	-	-	-	-	-	45.76	109.47

Parameter	Potential parameters values						
	Coulomb	van der Waals		Bond		Angle	
	$\frac{e^2}{4\pi\varepsilon_0}\sum_{i\neq j}\frac{q_i q_j}{r_{ij}}$	$\sum_{i\neq j} 4\varepsilon_{ij}\left(\left(\frac{\sigma_{ij}}{r_{ij}}\right)^{12}-\left(\frac{\sigma_{ij}}{r_{ij}}\right)^{6}\right)$		$k_1(r_{ij}-r_0)^2$		$k_2(\theta_{ijk}-\theta_0)^2$	
	$q_{i\ or\ j}$ (eV)	$\varepsilon_{i\ or\ j}$ (kcal/mol)	$\sigma_{i\ or\ j}$ (Å)	k_1	r_0	k_2	θ_0
Kaolinite ($Al_2Si_2O_5(OH)_4$)							
Al	1.575	1.3298e-6	4.2712	-	-	-	-
$Si_{b/h}$	2.100	1.840e-6	3.302	-	-	-	-
O_b	-1.050	0.15539	3.166	-	-	-	-
O_h	-0.950	0.15539	3.166	-	-	-	-
H_h	0.425	0.0000	0.000	-	-	-	-
O_h-H	-	-	-	-	-	15	110
Al-O_h-H	-	-	-	554.1349	1.0	-	-
Sodium Chloride (NaCl)							
Na^+	1.000	0.352642	2.424	-	-	-	-
Cl^-	-1.000	0.012785	5.422	-	-	-	-

S2.1Results of molecular dynamics simulation

After the water droplet reached equilibrium, the average water density profile was calculated over the production period. Stabilization was confirmed by tracking the normalized center of mass of water molecules as a function of time (**Figure S2**). Normalization was carried out using $COM_{Norm} = (COM - COM_{Min})/(COM_{Max} - COM_{Min})$, where COM denotes the center of mass, and the subscripts *Norm*, *Min*, and *Max* represent the normalized, minimum, and maximum center of mass values, respectively. The resulting two-dimensional density profile was converted into a binary image, and the contact angle was determined using the Low-Bond Axisymmetric Drop Shape Analysis (LBADSA) plugin in ImageJ [11,12].

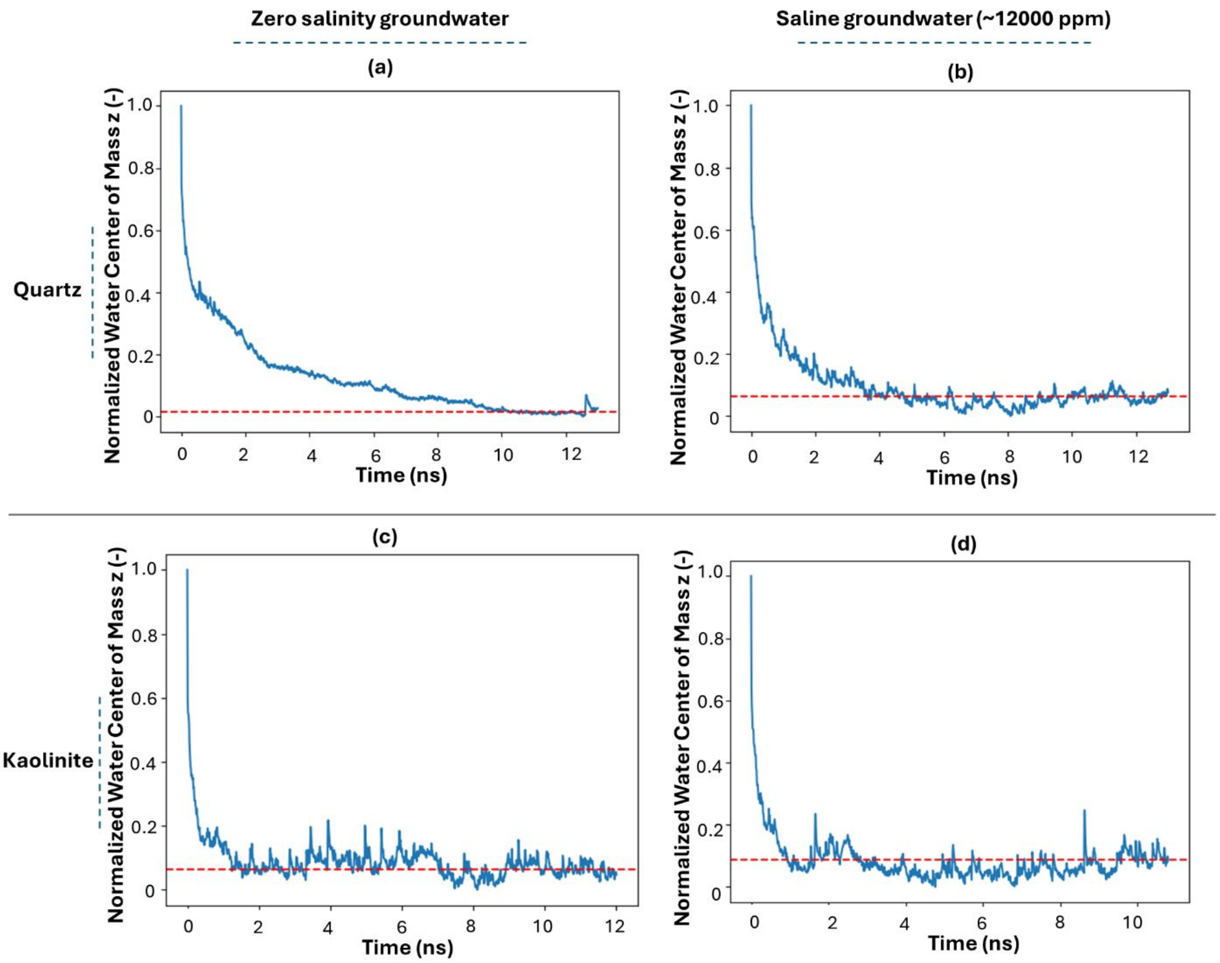

Figure S2. Normalized center of mass of water in the z-direction as a function of time (ns stands for nanoseconds), used to assess the stabilization of the water droplet: (a) Zero salinity groundwater-CH_4-quartz, (b) Saline groundwater-CH_4-quartz, (c) Zero salinity groundwater-CH_4-kaolinite, and (d) Saline groundwater-CH_4-kaolinite. The red dotted line indicates the average value during the production phase.

S2.2 Comparison of molecular dynamics simulation results with lab results

We compared the contact angle and its cosine for the quartz–water–CH_4 system obtained in this study with previously reported results. The MD-computed contact angles fall within the range of previously reported laboratory measurements for quartz, supporting the reliability of the approach (**Figure S3**). However, this experimental variability due to

differences in sample preparation, surface conditions, and measurement techniques introduces uncertainty that should be considered in the analysis. For kaolinite, however, direct comparisons are limited, as no laboratory or simulation results under conditions matching the pressure, temperature, and salinity ranges that we used are available. Nonetheless, indirect evidence supports the expectation of higher contact angles on kaolinite surfaces. For example, Pan et al. (2019) reported that clay-coated quartz exhibited significantly higher contact angles than clean quartz, with values increasing from 0–5° to approximately 18° [13]. Similarly, in the work reported by Ali et al., although the simulations were performed at elevated pressures and high salinities, the contact angle was reported to be around 100° [14]. Taken together, these studies suggest that substituting quartz with kaolinite leads to an overall increase in the contact angle, consistent with our interpretation.

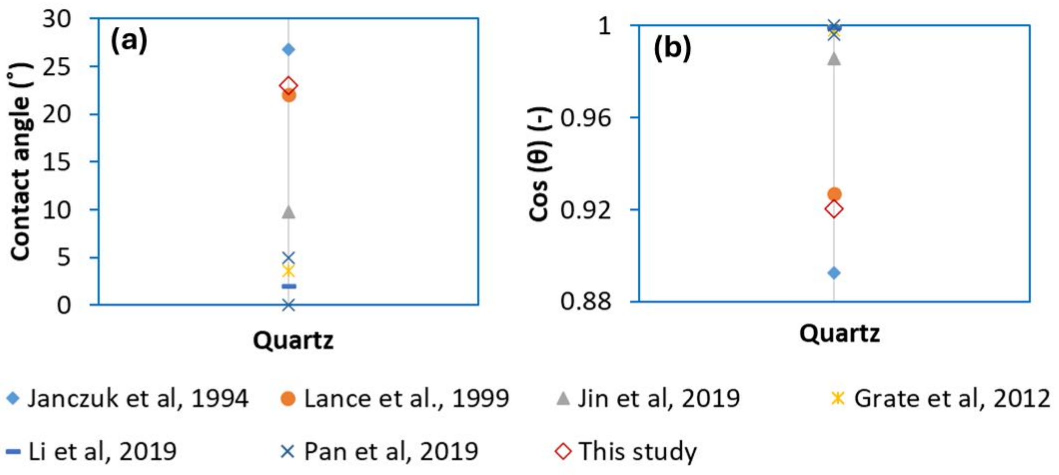

Figure S3. Comparison of estimated (a) contact angle values and (b) cosine of contact angle for a water-CH_4-quartz system with lab results [13,15–20].

S3 Conceptual model

Figure S4 illustrates the conceptual model developed to estimate CH_4 leakage. The system is divided into three regions including aquifer, vadose zone, and atmosphere to represent the leakage pathway. The aquifer and vadose zone are composed of two types of sediment: coarse-grain (CG, represented by quartz) and fine-grain (FG, represented by kaolinite). The aquifer is assumed to be fully water-saturated, the vadose zone partially saturated, and the atmosphere fully air-saturated. Relative permeability curves for all sections and the capillary pressure curve for the aquifer are shown in **Figure S5**. The initial pressure is specified for each section. Once the simulation begins, the water stream creates a pressure gradient across the aquifer, with an average pressure of approximately 1.7 bar, compared to about 1 bar in the vadose zone and the atmosphere. The capillary pressure curve in the vadose zone was the same as aquifer values.

We assume identical water relative permeability curves during both gas invasion (drainage) and subsequent water redistribution (imbibition), consistent with the predominantly water-wet nature of the system considered. In contrast, the gas (CH_4) phase exhibits relative permeability hysteresis, with its flow behavior depending on whether methane is displacing water during leakage (drainage) or being displaced by water during post-leakage redistribution (imbibition). Accounting for gas-phase hysteresis is essential for accurately representing residual trapping and snap-off mechanisms, which directly control the persistence and mobility of leaked methane [21].

Previous studies have demonstrated that neglecting relative permeability hysteresis can lead to significant misestimation of residual gas saturation and mobile gas volumes [21]. During imbibition, hysteresis reduces CH_4 mobility, thereby enhancing capillary trapping

and limiting further upward migration. Owing to the lack of experimental hysteresis data for the CH_4–water system under the conditions considered in this study, we adopt the widely used Land model [22] to represent gas-phase relative permeability hysteresis. Land's model is based on gas–water experimental data and is therefore representative of strongly water-wet conditions, which is consistent with the aquifer setting considered in this study [22]. Capillary pressure–saturation and relative permeability–saturation relationships are defined based on the Brooks–Corey correlation, with model parameters calibrated using core-flooding experimental data [23,24].

In the simulator, gas relative permeability initially follows the primary drainage curve. When flow reverses from drainage to imbibition, the model computes the residual (trapped) gas saturation associated with that reversal point using Land's correlation. At the onset of imbibition, the historical maximum gas saturation S_{gh} is recorded for each grid cell. The corresponding residual gas saturation during imbibition, S_{grh}, is then calculated from Land's equation using the specified maximum residual gas saturation $S_{gr}^{\max}$ (It is 0.3 in our simulation.). This relationship ensures that higher values of S_{gh} result in larger amounts of trapped gas, consistent with experimental observations for strongly water-wet systems.

Once S_{grh} is determined, the simulator constructs the imbibition gas relative permeability curve by shifting the drainage curve toward lower effective gas saturation. Specifically, during imbibition the gas relative permeability is evaluated as

$$k_{r,g}^{\text{imb}}(S_g) = k_{r,g}^{\text{dr}}(S_g^{\text{shifted}}),$$

where the shifted gas saturation S_g^{shifted} is a function of the current gas saturation S_g, the historical maximum saturation S_{gh}, and the trapped gas saturation S_{grh}. This shifting procedure preserves the shape of the drainage curve while enforcing residual trapping during imbibition.

Mass transfer between gas and aqueous phases is handled through equilibrium partitioning based on Henry's law [25], with pressure-dependent corrections where appropriate. Diffusive transport in the aqueous phase is also included, while reactive processes are neglected in this study to isolate the role of wettability and capillary heterogeneity on flow and trapping behavior.

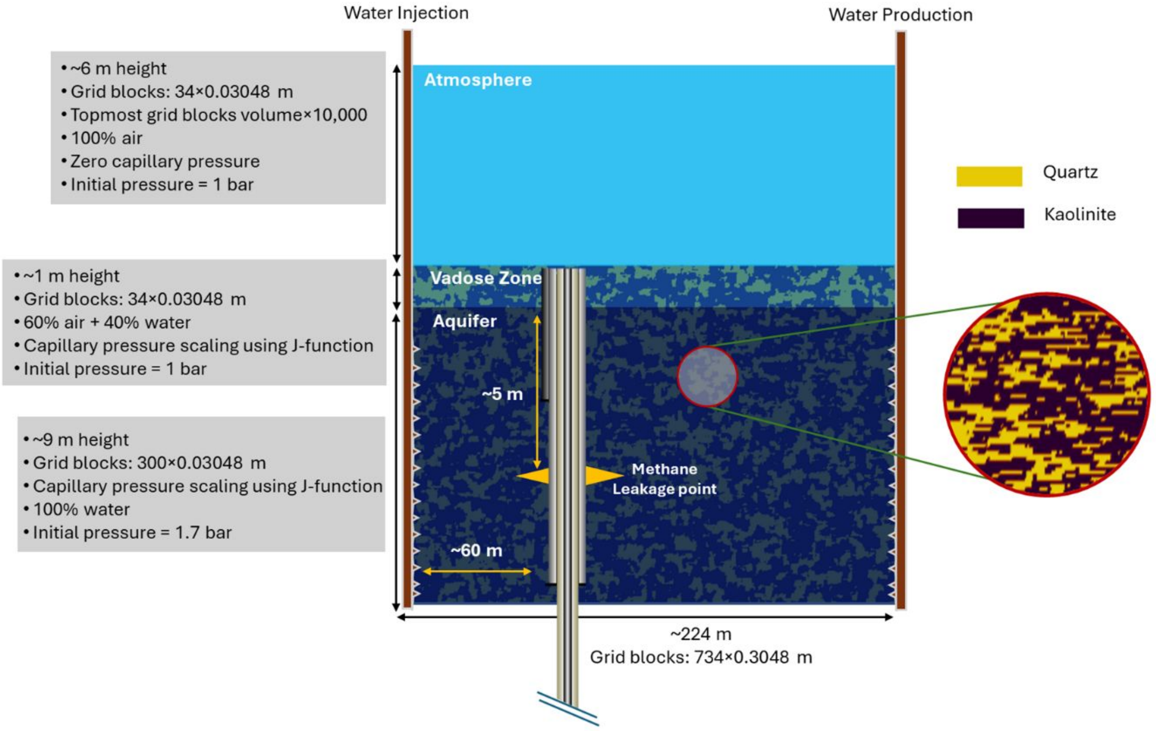

Figure S4. Conceptual model of CH_4 plume propagation from the leakage point and its migration from the aquifer to vadose zone, and atmosphere. Coarse-granular (quartz) and fine-granular (kaolinite) facies are distributed in the aquifer and vadose zone. The top atmospheric layer is scaled by 10,000 to provide adequate volume for CH_4 leakage.

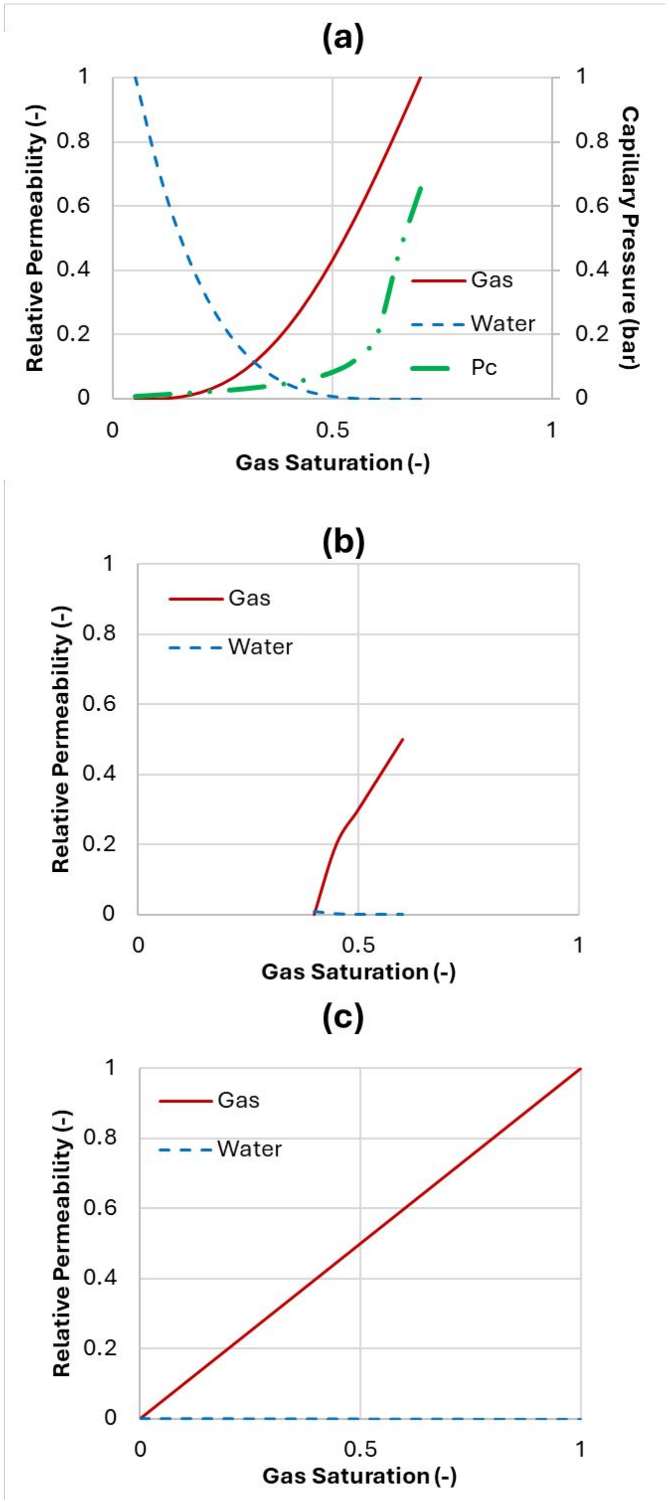

Figure S5. Relative permeability and capillary pressure curves for each section: (a) Aquifer, (b) vadose zone and (c) atmosphere. Capillary pressure is explicitly defined for the aquifer (P_c), while the vadose zone uses a scaled version. Capillary effects in the atmosphere are assumed negligible.

S3.1Fluid properties

Fluid properties are modelled using Peng-Robinson equation of state [26]. In the simulations, CH_4 is assumed to be soluble in water, whereas air is treated as an insoluble gas. Separate fluid models were calibrated for both air and CH_4, and the resulting property data were integrated into numerical simulations. Air is treated as a pseudo-pure fluid, and its viscosity and density were selected as the primary tuning parameters using experimental data from Lemmon et al (2004) [27]. In the case of CH_4, viscosity, density, and solubility in water were adjusted during the calibration process using experimental data from Lekvam and Bishnoi (1997) [28]. The resulting fluid properties obtained from the tuned models are summarized in **Table S2**.

Table S2. Fluid properties of air and CH_4 after fluid model tuning on lab results [27,28]

No.	Property (Unit)	Value	
		**Air**	**Methane (CH_4)**
1	Specific gravity (-)	1.0	0.3
2	Critical pressure (bar)	45.4	37.4
3	Critical temperature (K)	132.6	190.6
4	Acentric factor (-)	8.796e-3	8e-3
5	Molecular weight (g/mol)	28.96	16.04
Solubility			
6	Reference Henry's constant (bar)	-	387912
7	Reference pressure (bar)	-	14.7
8	Infinity volume (l/mol)	-	3.5e-2

S3.2Sediments properties

Table S3 summarizes the statistical parameters used to generate 25 stochastic realizations of CG and FG facies distributions. The realizations were constructed using the transition-probability Markov chain approach implemented in T-PROGS [29], which

ensures geologically consistent facies architectures using volumetric proportions and directional transition probabilities. For each facies, the mean and variance of permeability are also reported. As porosity and permeability are strongly correlated, porosity was derived from permeability using the following correlation [23,30]:

$$\phi = \left(\frac{k}{10^8}\right)^{0.1} \quad (1)$$

where k is permeability in mD, and ϕ is porosity in fraction.

Table S3. Facies classification and associated univariate statistical properties of the permeability distribution: Volumetric proportions of each facies type, their mean lengths in the horizontal and vertical directions (in meters), and the statistical characteristics of permeability (in mD), including the mean (k_{avg}) and variance (σ_k^2) of the natural logarithm of permeability, data partly taken from Ershadnia et al. (2020) and Ramanathan et al. (2010) [21,31].

**Facies types**	***Volume proportion (-)***	***Horizontal mean length (m)***	***Vertical mean length (m)***	***ln (k_{avg}) (mD)***	***ln (σ_k^2) (mD^2)***
Coarse grain (CG), rich in Quartz	0.39	3.00	0.18	8.5	0.3
Fine grain (FG), coated with Kaolinite	0.61	5.85	0.30	6.21	0.3

S4 Results of continuum-scale simulations

S4.1Quantitative distribution of methane

Figure S6 shows the converged distribution of CH_4 after 190 days of simulation for the base scenario, averaged across 25 stochastic realizations. Results are presented for the aquifer, vadose zone, and atmosphere, with CH_4 further divided into mobile, snap-off trapped, and dissolved phases in the aquifer.

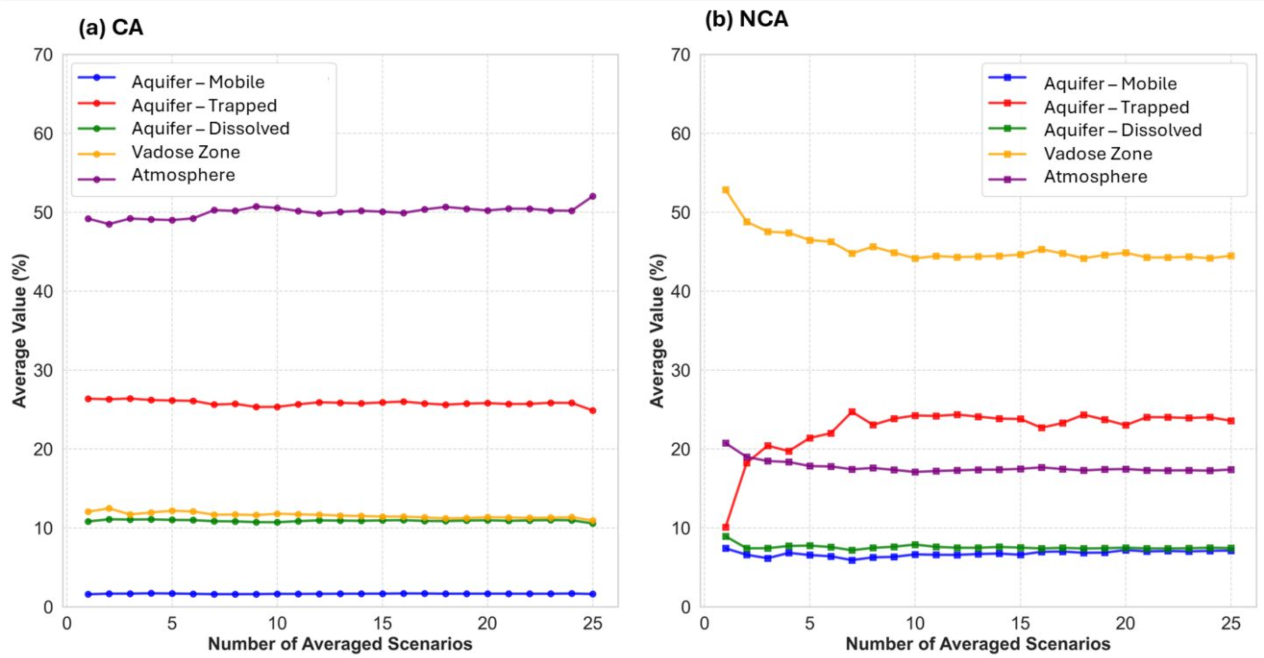

Figure S6. Converged quantitative distribution of averaged CH_4 % in different realizations across different sections and phases for the Base Scenario after 190 days of simulation, based on 25 realizations. Abbreviations: CA = contact angle included in capillary pressure scaling; NCA = no contact angle included**.**

S4.2Uncertainty in contact angle

As contact angles can vary due to impurities, surface heterogeneity, and other uncontrolled factors in natural systems, we introduced an uncertainty of ±10° to account

for this variability; see **Figure S7**. To evaluate its impact, we considered two extreme scenarios: (1) a maximum-contrast case in which quartz had a low contact angle of 10° and kaolinite a high angle of 73°, and (2) a minimum-contrast case in which quartz had a higher angle of 30° and kaolinite a lower angle of 53°. These scenarios were compared to a baseline case with fixed contact angles. The results, shown in Figure S8, indicate that incorporating this range of contact angle variability changed CH_4 leakage to the atmosphere by approximately 20% in the minimum-contrast case, and up to 40% in the maximum-contrast case. In other words, the uncertainty in contact angle alone can introduce about a 10% variation in predicted CH_4 leakage, highlighting its significant role in controlling leakage behavior and the importance of accurately constraining this parameter in modeling studies.

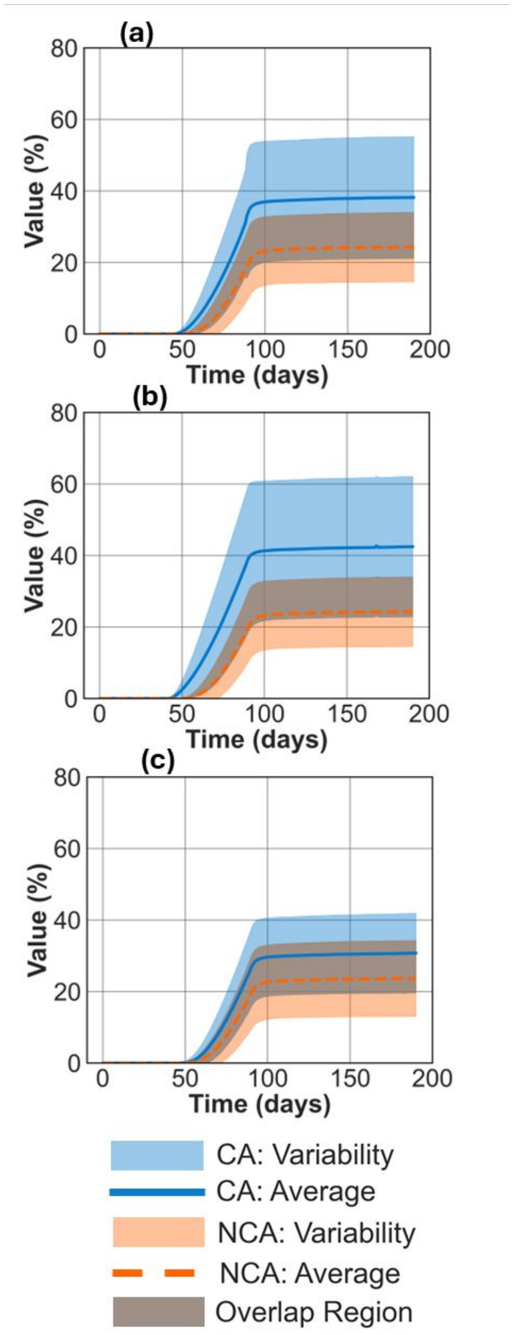

Figure S7. Atmospheric CH_4 leakage under three scenarios reflecting uncertainty in contact angles: (a) Base case, (b) Maximum contrast (Quartz: 10°, Kaolinite: 73°), and (c) Minimum contrast (Quartz: 30°, Kaolinite: 53°).

S4.3Methane in vadose zone

Since CH_4 amount in the vadose zone is lower than in the aquifer and atmospheric sections, the main text reports only the total CH_4. Here, we provide an example from a representative scenario of mineral distribution to illustrate the fraction of trapped CH_4 relative to the total in the vadose zone, as shown in **Figure S8**. Nearly 80% of CH_4 is retained due to hysteresis either in CA or NCA scenarios. The other important observation is that the rate of CH_4 concentration increase in CA is greater than NCA which is mostly due to new pathways along the FG facies.

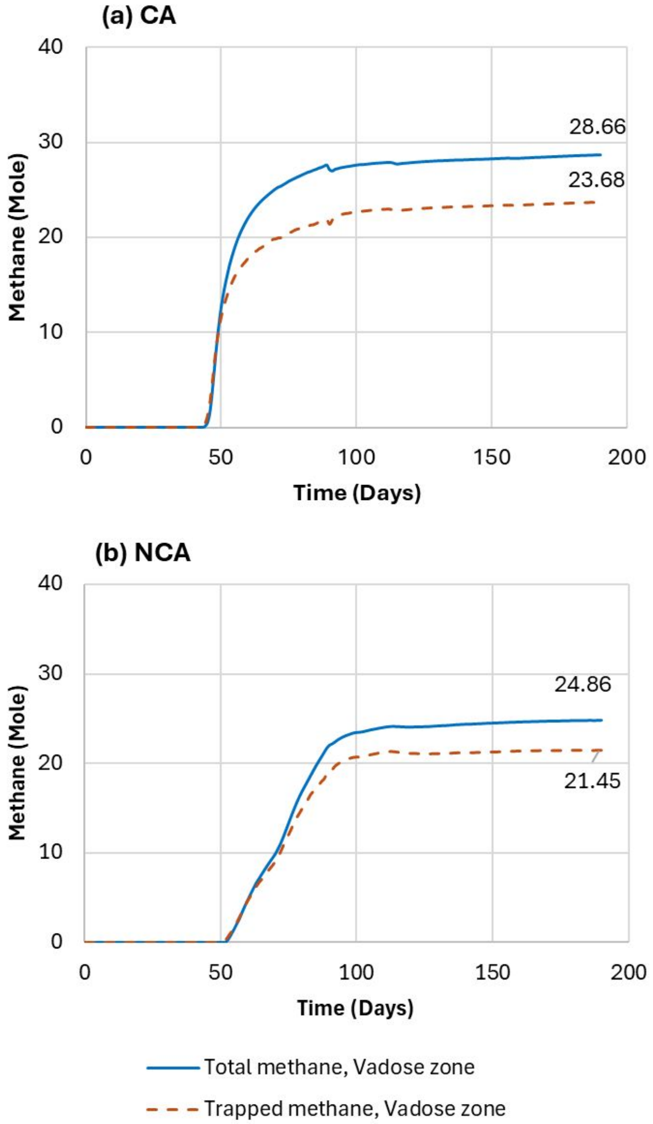

Figure S8. Comparison of trapped versus total CH_4 in the vadose zone for a representative mineral distribution realization: (a) with contact angle effects included (CA), (b) without considering contact angle effects (NCA)

References

(1) Boruvka, L.; Neumann, A. W. Generalization of the Classical Theory of Capillarity. *J. Chem. Phys.* **1977**, *66* (12), 5464–5476.

(2) Kanduč, M. Going beyond the Standard Line Tension: Size-Dependent Contact Angles of Water Nanodroplets. *J. Chem. Phys.* **2017**, *147* (17), 1.

(3) Berendsen, H. J. C.; Grigera, J.-R.; Straatsma, T. P. The Missing Term in Effective Pair Potentials. *Journal of Physical Chemistry* **1987**, *91* (24), 6269–6271.

(4) Pouvreau, M.; Greathouse, J. A.; Cygan, R. T.; Kalinichev, A. G. Structure of Hydrated Gibbsite and Brucite Edge Surfaces: DFT Results and Further Development of the ClayFF Classical Force Field with Metal–O–H Angle Bending Terms. *The Journal of Physical Chemistry C* **2017**, *121* (27), 14757–14771.

(5) Cygan, R. T.; Greathouse, J. A.; Kalinichev, A. G. Advances in Clayff Molecular Simulation of Layered and Nanoporous Materials and Their Aqueous Interfaces. *The Journal of Physical Chemistry C* **2021**, *125* (32), 17573–17589.

(6) Jorgensen, W. L.; Maxwell, D. S.; Tirado-Rives, J. Development and Testing of the OPLS All-Atom Force Field on Conformational Energetics and Properties of Organic Liquids. *J. Am. Chem. Soc.* **1996**, *118* (45), 11225–11236. https://doi.org/10.1021/JA9621760/SUPPL_FILE/JA11225.PDF.

(7) Joung, I. S.; Cheatham III, T. E. Determination of Alkali and Halide Monovalent Ion Parameters for Use in Explicitly Solvated Biomolecular Simulations. *J. Phys. Chem. B* **2008**, *112* (30), 9020–9041.

(8) Nosé, S. A Unified Formulation of the Constant Temperature Molecular Dynamics Methods. *J. Chem. Phys.* **1984**, *81* (1), 511–519.

(9) Zheng, R.; Germann, T. C.; Gross, M.; Mehana, M. Molecular Insights into the Impact of Surface Chemistry and Pressure on Quartz Wettability: Resolving Discrepancies for Hydrogen Geo-Storage. *ACS Sustain. Chem. Eng.* **2024**, *12* (14), 5555–5563.

(10) Zheng, R.; Germann, T. C.; Huang, L.; Mehana, M. Driving Mechanisms of Quartz Wettability Alteration under In-Situ H2 Geo-Storage Conditions: Role of Organic Ligands and Surface Morphology. *Int. J. Hydrogen Energy* **2024**, *59*, 1388–1398.

(11) Abràmoff, M. D.; Magalhães, P. J.; Ram, S. J. Image Processing with ImageJ. *Biophotonics international* **2004**, *11* (7), 36–42.

(12) Stalder, A. F.; Melchior, T.; Müller, M.; Sage, D.; Blu, T.; Unser, M. Low-Bond Axisymmetric Drop Shape Analysis for Surface Tension and Contact Angle Measurements of Sessile Drops. *Colloids Surf. A Physicochem. Eng. Asp.* **2010**, *364* (1–3), 72–81.

(13) Pan, B.; Jones, F.; Huang, Z.; Yang, Y.; Li, Y.; Hejazi, S. H.; Iglauer, S. Methane (CH4) Wettability of Clay-Coated Quartz at Reservoir Conditions. *Energy & fuels* **2019**, *33* (2), 788–795.

(14) Ali, A.; Cole, D. R.; Striolo, A. Cushion Gas Effects on Clay-Hydrogen-Brine Wettability at Conditions Relevant to Underground Gas Storage. *Int. J. Hydrogen Energy* **2024**, *58*, 668–677.

(15) Jin, J.; Wang, X.; Wick, C. D.; Dang, L. X.; Miller, J. D. Silica Surface States and Their Wetting Characteristics. *Surf. Innov.* **2020**, *8* (3), 145–157.

(16) Grate, J. W.; Dehoff, K. J.; Warner, M. G.; Pittman, J. W.; Wietsma, T. W.; Zhang, C.; Oostrom, M. Correlation of Oil–Water and Air–Water Contact Angles of Diverse Silanized Surfaces and Relationship to Fluid Interfacial Tensions. *Langmuir* **2012**, *28* (18), 7182–7188.

(17) Yu, T.; Li, Q.; Hu, H.; Tan, Y.; Xu, L. Molecular Dynamics Simulation of the Interfacial Wetting Behavior of Brine/Sandstone with Different Salinities. *Colloids Surf. A Physicochem. Eng. Asp.* **2022**, *632*, 127807.

(18) Janczuk, B.; Zdziennicka, A. A Study on the Components of Surface Free Energy of Quartz from Contact Angle Measurements. *J. Mater. Sci.* **1994**, *29* (13), 3559–3564.

(19) Yu, T.; Li, Q.; Hu, H.; Tan, Y.; Xu, L. Molecular Dynamics Simulation of the Interfacial Wetting Behavior of Brine/Sandstone with Different Salinities. *Colloids Surf. A Physicochem. Eng. Asp.* **2022**, *632*, 127807.

(20) Eske, L. D.; Galipeau, D. W. Characterization of SiO2 Surface Treatments Using AFM, Contact Angles and a Novel Dewpoint Technique. *Colloids Surf. A Physicochem. Eng. Asp.* **1999**, *154* (1–2), 33–51.

(21) Ershadnia, R.; Wallace, C. D.; Hosseini, S. A.; Dai, Z.; Soltanian, M. R. Capillary Heterogeneity Linked to Methane Lateral Migration in Shallow Unconfined Aquifers. *Geophys. Res. Lett.* **2021**, *48* (23), e2021GL095685.

(22) Land, C. S. Calculation of Imbibition Relative Permeability for Two-and Three-Phase Flow from Rock Properties. *Society of Petroleum Engineers Journal* **1968**, *8* (02), 149–156.

(23) Khandoozi, S.; Shik Han, W.; Kim, K.-Y.; Dai, Z.; Mehana, M.; Cole, D. R.; Reza Soltanian, M. Enhancing Predictive Understanding and Accuracy in Geological Carbon Dioxide Storage Monitoring: Simulation and History Matching of Tracer Transport Dynamics. *Chemical Engineering Journal* **2024**, 153127. https://doi.org/10.1016/J.CEJ.2024.153127.

(24) Brooks, R. H.; Corey, A. T. Properties of Porous Media Affecting Fluid Flow. *Journal of the irrigation and drainage division* **1966**, *92* (2), 61–90.

(25) Smith, F. L.; Harvey, A. H. Avoid Common Pitfalls When Using Henry's Law. *Chem. Eng. Prog.* **2007**, *103* (9), 33–39.

(26) Robinson, D. B.; Peng, D.-Y. *The Characterization of the Heptanes and Heavier Fractions for the GPA Peng-Robinson Programs*; Gas processors association, 1978.

(27) Lemmon, E. W.; Jacobsen, R. T. Viscosity and Thermal Conductivity Equations for Nitrogen, Oxygen, Argon, and Air. *Int. J. Thermophys.* **2004**, *25*, 21–69.

(28) Lekvam, K.; Bishnoi, P. R. Dissolution of Methane in Water at Low Temperatures and Intermediate Pressures. *Fluid Phase Equilib.* **1997**, *131* (1–2), 297–309.

(29) Carle, S. F. T-PROGS: Transition Probability Geostatistical Software, Version 2.1. *Department of Land, Air and Water Resources, University of California, Davis* **1999**.

(30) Abedini, A.; Torabi, F. Pore Size Determination Using Normalized J-Function for Different Hydraulic Flow Units. *Petroleum* **2015**, *1* (2), 106–111. https://doi.org/10.1016/J.PETLM.2015.07.004.

(31) Ramanathan, R.; Ritzi Jr, R. W.; Allen-King, R. M. Linking Hierarchical Stratal Architecture to Plume Spreading in a Lagrangian-based Transport Model: 2. Evaluation Using New Data from the Borden Site. *Water Resour. Res.* **2010**, *46* (1).
